# Clinical laboratory validation of the MCL35 assay for molecular risk stratification of mantle cell lymphoma

**DOI:** 10.1007/s12308-020-00418-4

**Published:** 2020-10-13

**Authors:** Colleen A. Ramsower, Alanna Maguire, Ryan S. Robetorye, Andrew L. Feldman, Sergei I. Syrbu, Allison C. Rosenthal, Lisa M. Rimsza

**Affiliations:** 1 Department of Laboratory Medicine & Pathology, Mayo Clinic Arizona, 13400 E. Shea Blvd, CRB1-263, Scottsdale, AZ 85259 USA; 2 Department of Research, Mayo Clinic Arizona, Scottsdale, AZ USA; 3 Department of Laboratory Medicine and Pathology, Mayo Clinic Minnesota, Rochester, MN USA; 4 Department of Pathology, University of Iowa, Iowa City, IA USA; 5 Internal Medicine, Division of Hematology/Oncology, Mayo Clinic Arizona, Scottsdale, AZ USA

**Keywords:** Mantle cell lymphoma, Gene expression profiling, Ki67, Prognosis

## Abstract

Mantle cell lymphoma (MCL) is a clinically heterogeneous B cell malignancy for which a variety of prognostic factors have been proposed. Previously, a digital gene expression profiling “proliferation signature” capable of risk stratifying MCL was identified and subsequently developed into a multi-analyte prognostic assay, known as the “MCL35” assay. In this study, we sought to explore the performance characteristics of the MCL35 assay in a clinical laboratory and compare results with the Ki67 proliferation marker. The results describe the clinical validation of the MCL35 assay for molecular risk stratification of MCL including accuracy, sensitivity, specificity, use in acid-decalcified bone marrow core biopsies, fixatives, lower limit of RNA input, quality metrics, and other laboratory parameters. The resulting data indicate that this is a robust technique with outstanding reproducibility. Overall, the data support the concept of molecular signatures, as assessed with digital gene expression profiling, for improved standardization and reproducibility for proliferation assessment in MCL.

**Electronic supplementary material:**

The online version of this article (10.1007/s12308-020-00418-4) contains supplementary material, which is available to authorized users.

## Introduction

Mantle cell lymphoma (MCL) is a B cell malignancy that accounts for 2–6% of all non-Hodgkin B cell malignancies and is recognized by the World Health Organization (WHO) as a unique entity whose optimal clinical management is evolving [1]. With rare exceptions, MCL is characterized by a chromosomal translocation at t(11;14)(q13;q32) that relocates the CCND1 gene from its position at 11q13 to 14q32, placing it adjacent to the highly transcribed immunoglobulin heavy chain gene (IGH), resulting in overexpression of Cyclin D1, the downstream protein product of CCND1, and a potent driver of cell proliferation [2]. Further underscoring the importance of Cyclin D1 and proliferation in MCL, point mutations and genomic deletions in CCND1 resulting in more stable Cyclin D1 transcripts with extended half-lives are associated with reduced survival [3].

Despite the emergence of novel agents that have substantially improved MCL outcomes, there is no universally accepted standard of care for MCL and therapeutic responses remain highly variable [1]. Treatment regimen heterogeneity likely stems from general guidelines based on the patient’s health and perceived ability to tolerate regimens (predominantly age). Attempts to stratify MCL patients into risk groups have shown some promise, where the most commonly used stratification method in clinical practice to date is the MCL International Prognostic Index (MIPI). The MIPI relies on a combination of both clinical factors and lab results and has demonstrated reasonable stratification in clinical trial cohorts [4, 5]. Immunohistochemical staining for Ki67, the most widely used clinical marker of cell proliferation, is sometimes used as a surrogate marker for patient stratification and is believed to add value when used in conjunction with the MIPI; termed “biologic MIPI” or MIPI-b [6, 7]. However, methods utilizing clinical data, tumor morphology, and/or immunohistochemistry are highly subject to inter-observer and inter-laboratory variability [8, 9].

Using gene expression profiling, the Lymphoma/Leukemia Molecular Profiling Project (LLMPP) research consortium identified a proliferation signature that molecularly stratifies MCL patients into low-, standard, and high-risk groups that were significantly associated with overall survivals of 1.1, 2.6, and 8.6 years, respectively (log-rank for trend *p* < .001) [10]. Originally discovered using targeted “LymphoChip” microarrays, the proliferation signature was later enhanced using the Affymetrix GEP platform before being translated to the clinically compatible, 510(k) FDA cleared digital gene expression profiling nCounter® platform from NanoString, resulting in the development of the MCL35 molecular profiling assay [11]. The MCL35 assay is a 35 target gene expression risk profiling assay that nCounter® technology (NanoString Technologies, Seattle WA) and algorithmic classification to robustly assign FFPE tissue specimens from diagnosed MCL patients into one of three risk categories termed low, standard, and high risk that has immense potential to guide prospective clinical trials and targeted drug therapies.

The MCL35 includes 17 informative genes and 18 housekeeping genes. Of the 17 informative genes, 13 positively and 4 negatively correlated with proliferation (poor and good survival respectively). These encompassed transcription factors, cyclins, and cyclin-dependent kinases, as well as genes involved in chromatin condensation, spindle formation, and chromosome segregation [11].

Here we report on the evaluation of the MCL35 assay as a clinical biomarker in the CAP-accredited/CLIA-certified Molecular Diagnostics-Arizona Laboratory (MDAZL), a lab with extensive experience in the development (Lymph2Cx, Lymph3Cx) and FDA validation (LymphMark™) of similar nCounter®-based molecular profiling assays [12]. Performance specifications for accuracy, precision, sensitivity, specificity, and clinical utility were assessed in this study.

## Materials and methods

FFPE tissues were obtained via the Mayo Clinic Molecular Epidemiology Resource (MER). Briefly, banked tissues meeting the criteria of a diagnosis of MCL were identified, and the tissue blocks reviewed for tissue content by a hematopathologist. Blocks with sufficient tissue remaining were sectioned at 4–5 μm, one reserved for hematoxylin and eosin (H&E) staining at the PRC. Upon arrival in Arizona, one section was stained with Ki67 per local laboratory protocols (described below).

The H&E stained slide was reviewed by an expert hematopathologist who identified a region containing 60% or more tumor by area and also recorded the tumor morphology: classic, pleomorphic, or blastoid. RNA was isolated using either the AllPrep DNA/RNA FFPE kit (Qiagen, Germantown, MD) or the HighPure FFPET RNA Isolation kit (Roche, Indianapolis, IN). The extracted RNA was quantitated using the NanoDrop and 200 ng total RNA was used as input in the nCounter Elements hybridization reaction. The nCounter® FLEX system purifies the reactions and counts the number of molecules of each gene’s mRNA transcript in each sample. The raw count data was assessed for quality control by the nSolver™ software version 3.0, which examined the linearity of synthetic positive controls and background levels of synthetic negative controls in each well. Once run quality was verified, the raw counts were processed through the locked stratification algorithm housed locally. If average counts for the housekeeping genes were < 80, the sample was deemed “Poor Quality”, and no further analysis was reported. The data that pass this QC step were normalized to the housekeeping genes before calculating a model score, which is used to stratify each case into a risk category. Samples scored accordingly: low risk (score less than − 143.6), standard risk (score of − 143.6 to less than − 27.6), and high risk (score greater than or equal to − 27.6).

### Synthetic positive controls

Ultra-pure oligos with an RNA backbone, or Ultramers (Integrated DNA Technologies, Coralville, IA), were procured to use as a positive control to be run on every cartridge. Ultramers matching the targeted RNA sequence for each gene in the MCL35 assay were pooled in equimolar amounts and aliquoted for single use. While not part of the algorithmic calculation or score adjustment, overall probability scores generated by the Ultramers were monitored for fluctuation across cartridges and reagent lots.

### Chemistry comparison

We initially performed a chemistry comparison between NanoString Technologies’ (Seattle, WA) “Standard XT” and “Elements XT” chemistries (hereafter referred to as “Standard” and “Elements”, respectively). In the former, pre-labeled probes are purchased from NanoString, while in the latter, unlabeled probes are hybridized to both the target RNA of interest and “tags” pre-labeled with a standardized set of NanoString’s fluorescent barcodes. As in our previous work with the Lymph2Cx assay, the Elements chemistry was evaluated due to the ability of the local laboratory to standardize the fluorescent barcodes between each run [12]. For this comparison, RNA from 12 specimens previously assayed with Standard chemistry in our prior MCL35 publication, representing the range of possible MCL35 risk scores, were assessed with Elements chemistry to examine for bias.

### Accuracy

A blinded study was performed to compare the MCL35 assay as performed in the MDAZL to the MCL35 results obtained in the published literature. Cases from all three risk categories (low, standard, high) were included. The MCL35 assay was performed on 25 FFPE previously characterized and published specimens, which included review by an expert panel of hematopathologists and which had matched fresh/frozen samples analyzed using the Affymetrix GEP platform. Additionally, the 12 samples from the chemistry comparison were considered part of the accuracy validation, as well as two MCL cell lines, Mino and Granta519. Since cell lines are designed to proliferate, it was expected that they should type into either the standard or high-risk groups. The cell lines were treated in the same manner as normal patient specimens, with cell pellets fixed in formalin and embedded in paraffin, one to five sections ranging in thickness from 4 to 10 μm used per extraction, and 200 ng total RNA input used in the assay. Risk scores and risk groups obtained from FFPE tissues by MDAZL were compared with the results in the literature of the matched fresh/frozen specimens processed with the Affymetrix GEP platform or Standard chemistry with NanoString.

### Precision

Intra- and inter-cartridge precision were assessed using both biological and technical replicates. To encompass all aspects of precision (across observers, cartridges, and days), six FFPE tissue specimens (two from each of the three risk categories) were utilized. All six specimens had RNA isolated independently in MDAZL by one technologist, and in addition, three of these six (one case from each risk category) were repeated from RNA isolation by a different technologist to represent true biological duplicates. Then, technical replicates (RNA from the same stock RNA sample processed multiple times with the assay) were processed by each respective technologist across multiple cartridges, days, and concentrations (ranging from 50 to 500 ng), for a total of 18 replicates for the three duplicated samples and 9 replicates for the non-duplicated cases over 13 cartridges and 8 days. MCL35 risk score and category was assigned to all specimens as previously described. Overall concordance with previously assigned risk categories from the GEP data was assessed for biological replicates, while the risk score in technical replicates were plotted to examine reproducibility and systematic bias.

### Pre-analytical variables

To evaluate the effect of different fixatives on assay performance, 11 cases with paired formalin-fixed and B5-fixed blocks were evaluated for RNA quality and MCL35 assay results. To evaluate the effect of different fixatives and decalcification on assay performance, we evaluated four formalin-fixed and decalcified, as well as six B5-fixed and acid-decalcified, bone marrow core biopsies. Since the original publication by Scott et al. [11] was performed only on lymph node biopsies (extra-nodal tissues were not included), we also sought to assess the performance of the MCL35 in 44 non-nodal tissues.

### Interfering substances

To evaluate the effects of interfering substances, RNA extracted from six specimens covering the spectrum of possible risk scores were spiked at 10% by volume in the hybridization reactions with DNA, a potentially cross-reactive contaminant, as well as potential chemical inhibitors that could carry through from RNA extraction (d-limonene, tissue lysis buffer RLT, column binding buffer FRN, and ethanol).

### Ki67 immunostaining and morphology

Whole tissue sections were deparaffinized on-instrument (Discovery Ultra, Roche, Indianapolis, IN). Heat-induced epitope retrieval was performed followed by primary antibody incubation at 37 °C for 32 min. Ki67 dispenser (Roche #790-4286 Clone 30-9) was visualized using Optiview DAB detection (Roche #760-700). Slides were counter stained with hematoxylin (Roche #760-2021) followed by bluing (Roche #760-2037), each for 4 min. H&E stains were performed manually. Scoring was performed by an experienced hematopathologist (LR) according to the best practices guidelines of the European Mantle Cell Lymphoma network [8]. The definition of agreement between the two methods was as follows: MCL35 low risk and Ki67 < 10%, MCL35 standard risk and Ki67 10% to < 30%, MCL35 high risk and Ki67 ≥ 30%. Tumor morphology was recorded as assessed by the hematopathologist during tissue review, with classic morphology representing a lower risk than pleomorphic or blastoid morphologies.

## Results

**Fig. 1:**
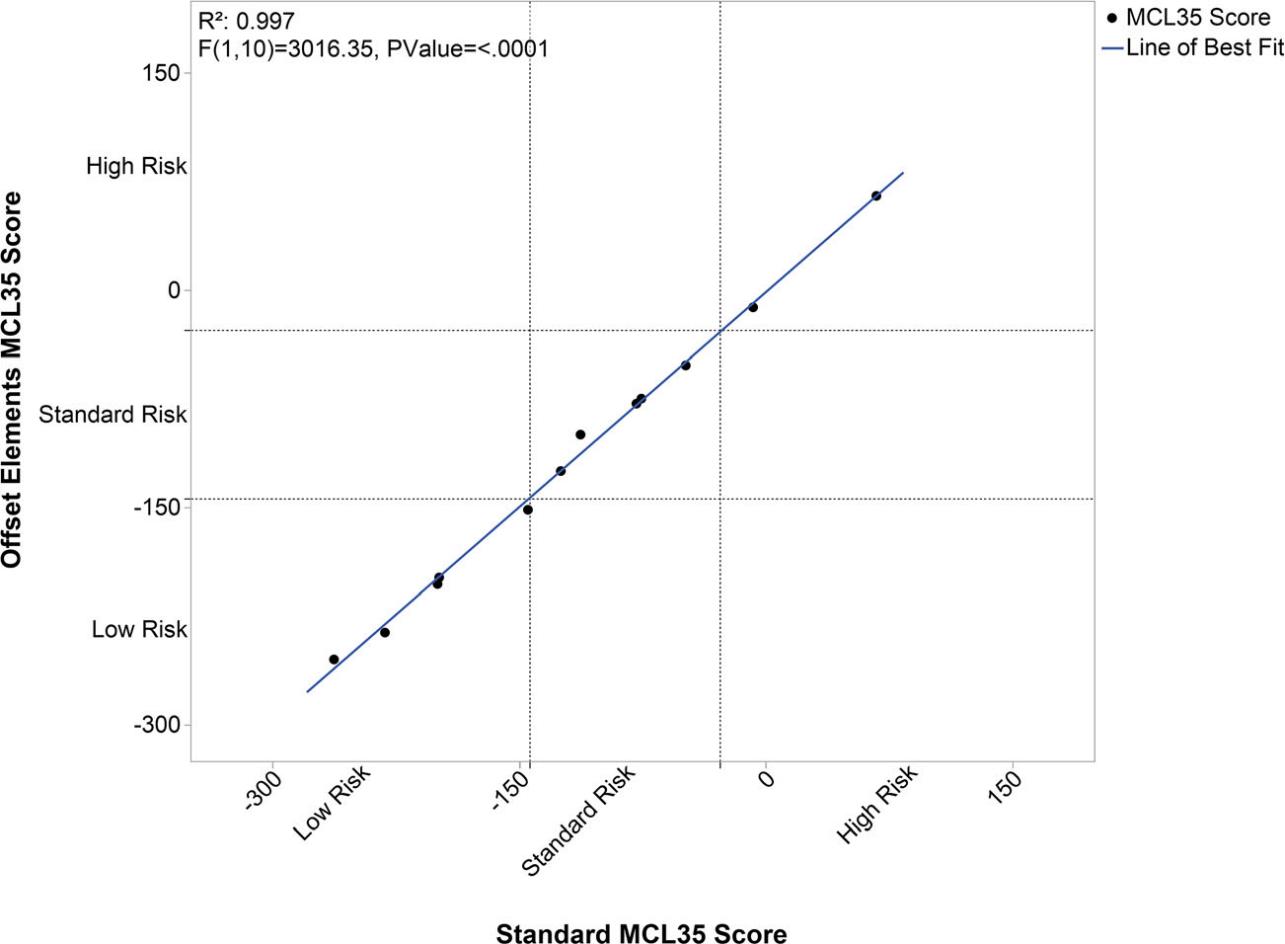
Scatter plot of the offset Elements MCL35 score vs the original Standard MCL35 score, with Spearman correlation of 0.997 (*p* < 0.0001) indicating no bias or attenuation between the chemistries

In a matched pairs analysis comparing the two different chemistry approaches, no attenuation based upon input or MCL35 score was observed; however, there was a systematic bias in the data, with Elements scores being an average of 22 points lower than the score generated by Standard chemistry (data not shown). An offset was incorporated into the MCL35 algorithm to add 22 points to scores generated with Elements chemistry, and upon re-processing the data through the offset algorithm, the Spearman correlation is excellent (0.997, *p* < 0.0001) with no attenuation (Fig. 1). All data presented herein was generated with Elements chemistry and therefore processed using the offset MCL35 algorithm.

To assess the accuracy of the MCL35 assay, 25 pretreatment MCL FFPE tumor specimens that had previously been characterized for MCL35 risk category by Affymetrix microarray using matched frozen material were assessed [11], where Affymetrix MCL scores and risk categorization generated using FF-derived RNA were considered the gold standard. Of the 25 samples examined, the two methods were 92% (23/25) concordant, with the two mismatches occurring in borderline cases. The MCL35 assay demonstrates a high level of accuracy (*R*^2^ = 0.971), while there is not significant attenuation.

**Fig. 2:**
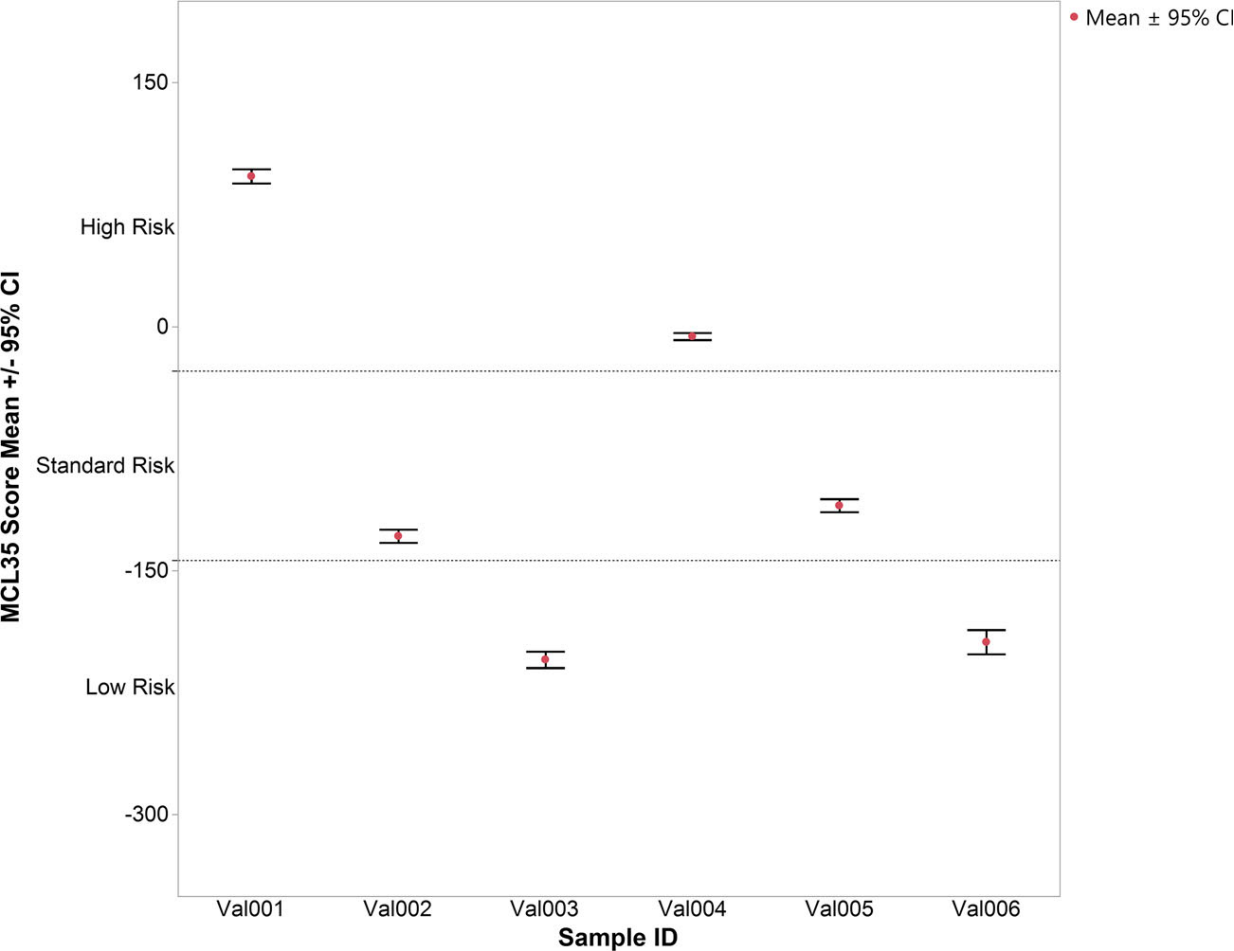
MCL35 precision data, plot of mean ± 95% CI for all replicates, and risk group cut points are shown to demonstrate that misclassification would only occur for borderline cases between adjacent risk groups

Precision was assessed as follows: six samples, two from each risk category, were assessed. Three of the six, one from each risk category, were re-extracted and run by a second user. Each technologist performed 9 technical replicates of each sample (except for sample Val002, which had one failure at a total input of 50 ng total RNA for a total of 8 replicates), across multiple cartridges and days and using variable inputs between 50 ng and 500 ng. Technical precision between users, cartridges, and days were outstanding; no replicate for any case was discordant with the original call, and the standard errors of the mean for all technical replicates for each sample are reported in Table 1. The maximum error was 3.2, which was above our original estimate of the maximum standard error; however, the preceding literature had fewer technical replicates and so may have underestimated the true variance. Since there is no gold standard for reference, in order to assess how the technical replicate spread could impact assay results, the mean of replicates for each precision sample was plotted with 95% confidence intervals (CI) using a *t* distribution for small sample numbers (Fig. 2). Since the mathematical distance between cut-points for the low/standard risk groups and standard/high-risk groups is 116 points, misclassification could only occur in borderline cases between adjacent risk groups.

**Table 1:** Precision data by technologist. Sample “Val002” had one failure at in input of 50 ng, resulting in a number of replicates *n* = 8

All replicates					
Sample ID	MCL35 risk group	*n*	Mean	Standard error	95% confidence interval
Val001	High	9	92.3	1.9	88.0 to 96.6
Val002	Standard	8	− 128.8	1.7	− 132.8 to − 124.7
Val003	Low	18	− 204.8	2.4	− 209.8 to − 199.8
Val004	High	18	− 6.0	1.0	− 8.2 to − 3.8
Val005	Standard	18	− 109.9	1.9	− 114.0 to − 105.9
Val006	Low	9	− 194.0	3.2	− 201.5 to − 186.5

Performance of the MCL35 assay with reduced RNA inputs was also investigated. Different RNA inputs (200 ng, 100 ng, 50 ng) were assessed for all samples from the precision cohort. The MCL35 assay demonstrated 100% concordance at 200 ng and 100 ng inputs. As previously mentioned, one sample from the precision cohort failed with a total RNA input of 50 ng, but the same sample yielded concordant risk group assignments across all other replicates. For small samples, such as core needle biopsies, routinely collected in clinical settings, reaching a total RNA input of 200 ng is quite reasonable while attaining a total RNA input of 500 ng would likely be unfeasible; thus, the recommended and lower RNA inputs for the MCL35 assay were established as 200 ng and 100 ng, respectively.

To evaluate MCL35 performance in non-formalin fixed samples, 11 different cases that had both formalin and B5 fixed tissue blocks were evaluated for RNA quality and assay results. Of these 11, both the formalin-fixed and B5-fixed blocks were “poor quality” per assay metrics in 2 cases. Of the remaining cases, 0/9 (0%) formalin-fixed cases were of poor quality, while 4/9 (44%) of the B5-fixed were of poor quality. Of the 5 cases with successful assays on both formalin-fixed and B5-fixed blocks, 4 resulted in the same risk group (1 low risk, 2 standard, 1 high risk), while 1 case was discrepant (low risk using the formalin-fixed block and standard risk using the B5-fixed block). This discrepancy could be a result of assaying different blocks (with slightly different tumor biology) or a result of fixation method. Adding non-paired samples as well to the paired samples, 17/29 (59%) of B5-fixed blocks failed compared with 9/36 (25%) of formalin-fixed blocks, thus showing a near doubling of assay failure with B5 fixation (*p* = 0.0061, one-tailed Fisher’s exact test for FFPE generating more passing results than B5). Overall, given the high failure rate, B5-fixed blocks are not preferred but may possibly provide an alternative tissue source in situations when no formalin fixed blocks are available.

To evaluate the usefulness of the MCL35 assay in bone marrow core biopsies, a frequent site of diagnosis, we evaluated samples fixed in formalin-fixed and acid decalcified (*n* = 4) or B5-fixed and decalcified (*n* = 6) blocks (*p* = 0.5714, 2-tailed Fisher’s exact test). Of the formalin-fixed, decalcified samples, only 1 in 4 yielded sufficient quality RNA for use in the assay at an input of 100 ng, while 3 of the 6 B5-fixed and decalcified samples yielded sufficient RNA. Although a small study, this indicates that decalcification appears to be detrimental to RNA retrieval.

Since the original publication by Scott et al. [11] was performed only on lymph node biopsies (extra-nodal tissues were not included), we also sought to assess the performance of the MCL35 in non-nodal tissues. In our analysis of 65 cases, we detected no difference in assay failure rate in nodal blocks (8/21 or 38%) versus non-nodal blocks (18/44 or 41%) (*p* = 1.0000, 2-tailed Fisher’s exact test). Clinical treatment information and patient outcome were not available on these retrospective cases to evaluate the prognostic power of the MCL35 in non-nodal samples. However, in a prior publication, the MCL35 risk assessment on 42 non-nodal samples (tonsils, gastrointestinal biopsies, soft tissue specimens, and other materials) was reported to correlate with overall survival [13].

The effect of spiking contaminants into the hybridization reaction was also explored. To determine if we could elicit an effect from contaminants, the reactions were spiked at 10% total volume during MCL35 validation. We chose contaminants that could potentially be carried through from the RNA extraction process, including d-Limonene deparaffinization solution, tissue lysis buffer RLT, column binding buffer FRN, 100% ethanol, and matched stock DNA, where the input DNA concentration ranged from 80.3 to 385.5 ng/μl. The volume of water in each nCounter reaction was reduced and replaced by 1.5 μl of a given contaminant, thereby ensuring that the final hybridization reaction volume (15 μl) and RNA input (200 ng) were kept constant. With zero mis-categorizations for all contaminants, sample Val003 appeared to be to particularly tolerant of all contaminants, suggesting that sample quality may play a role in contaminant tolerance. Proper adherence to RNA purification protocols in the laboratory ensure that contaminants do not carry through to assay performance.

**Fig. 3:**
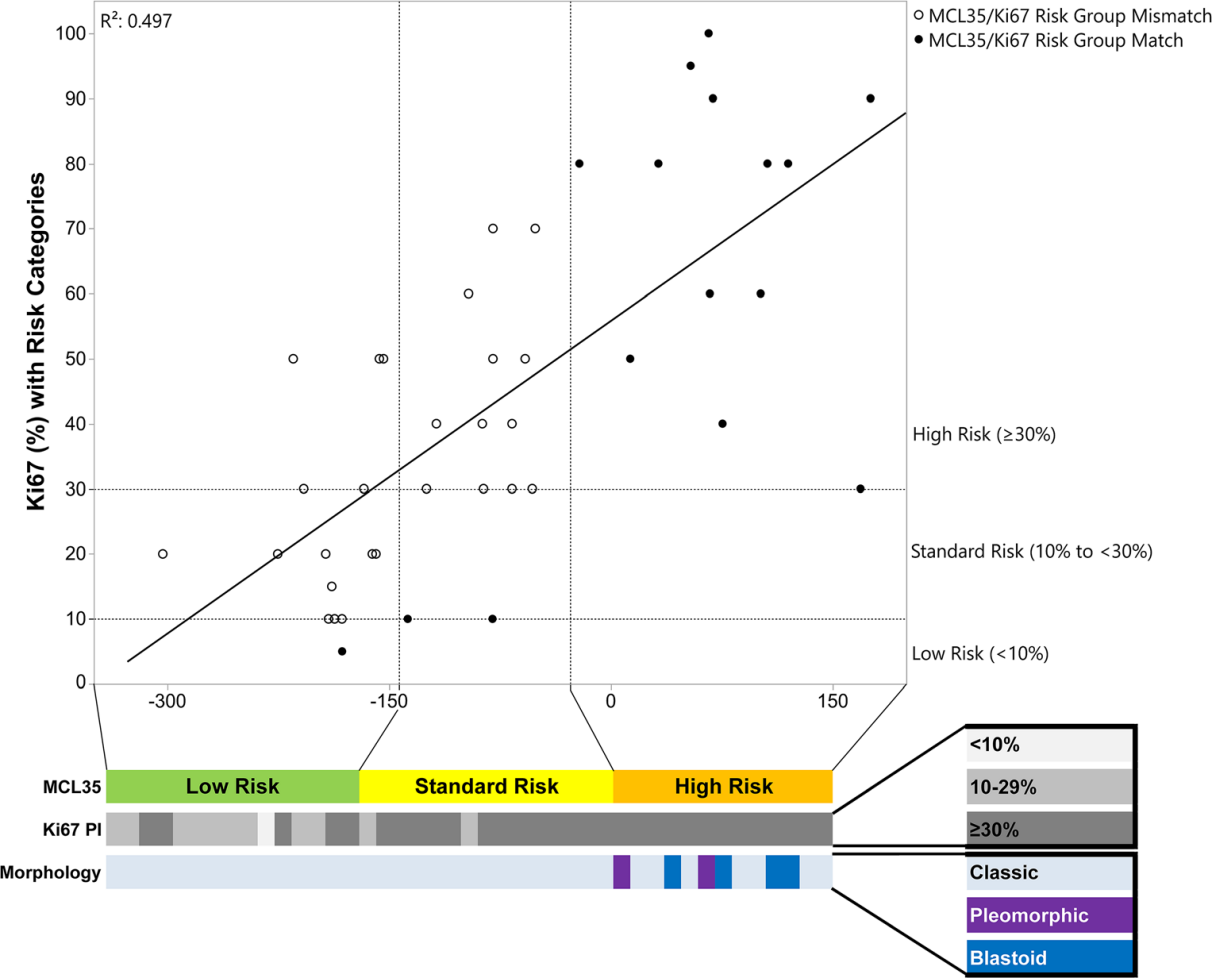
Scatter plot of Ki67 (%) vs MCL35 score with risk groups denoted for each assay and grouping of Ki67 and morphology by MCL35 score, indicating that while all high-risk patients are captured by all three methods, Ki67 proliferation index appears to overestimate risk when compared with MCL35

In comparison with Ki67 proliferation index and morphology, the percentage of tumor nuclei with Ki67 staining is a frequently used prognostic marker in MCL. This “proliferation index” is typically split into three groups: < 10%, 10% to < 30%, and ≥ 30% [6]. We analyzed a second series of (previously unpublished) MCL cases for both Ki67 and MCL35 (*n* = 43), and when compared, the Spearman correlation between the two methods was 0.497 (*p* < 0.0001). One case in this study had a Ki67 expression < 10%, 11 cases had expression of 10% to < 30%, and 31 cases had expression ≥ 30%. Using the MCL35 assay, all 13 cases denoted high risk were also high Ki67 expressers with a mean Ki67 expression of 72%, range 40–100%. The 15 cases denoted standard risk by the MCL35 assay had a mean Ki67 expression of 41%, range 10–70%; 14 of which had Ki67 expression ≥ 30%. Of the 15 cases denoted low risk by the MCL35 assay, one case had Ki67 expression < 10%, nine cases had expression of 10% to less than 30%, and 5 cases had expression ≥ 30%, with a mean Ki67 expression of 24%, range 5–50%. Overall, 16 of 43 (37%) of cases agreed between the two techniques (MCL35 low risk and Ki67 < 10%, MCL35 standard risk and Ki67 10% to < 30%, MCL35 high risk and Ki67 ≥ 30%), including all 13 cases of the high-risk group by MCL35, two cases of the standard risk group by MCL35, and one case from the low-risk group by MCL35. Given the current data, it appears that Ki67 estimates higher probability of risk overall than the MCL35 assay. All of the cases with the more aggressive morphologies (pleomorphic and blastoid) were classified as high risk by the MCL35 assay. All of this information is presented in Fig. 3.

Supplemental Table S1 summarizes all assay performance characteristics.

## Discussion

Herein, we describe the clinical validation of the FFPE-compatible nCounter-based digital gene expression MCL35 assay for risk stratification of MCL35 patients in a CAP/CLIA-certified clinical molecular diagnostics laboratory and comparison with Ki67 proliferation index. Of note, the MCL35 assay was not developed for use in leukemic, non-nodal MCL; however, a separate assay to identify that disease type was recently published and known as the L-MCL16 [14].

The MCL35 assay demonstrated excellent concordance with the gold standard signature defined by Affymetrix gene expression profiling thereby validating the accuracy of the assay. In terms of assay performance, samples run multiple times by different users clustered tightly together and yielded small standard errors indicating that the MCL35 assay is a high-precision assay. In addition, the MCL35 assay could robustly assign risk category from inputs as low as 50 ng of FFPE-derived RNA. The assay was also shown to be capable of correctly risk categorizing tumor samples in the presence of extraordinarily high levels of contaminants that would not likely occur in a CAP/CLIA-certified laboratory due to stringent handling. Standardized operating procedures have been written, and the assay was launched as a laboratory-developed test in the MDAZL with plans for use in retrospective analysis of clinical trial materials and emerging plans for use in prospective clinical trials.

Acid-decalcified core biopsies were successfully analyzed in only 4 of 10 cases attempted. Other methods of decalcification were not evaluated. In addition, we noted that all 4 successful core biopsies resulted in high-risk MCL35 scores. Caution is therefore advised in the use of bone marrow core biopsies for the MCL35 assay since the proliferation of the background hematopoietic cells is so high that it may dominate the results. In this study, we could not evaluate the prognostic power of the MCL35 in other types of non-nodal samples due to lack of clinical annotation. However, in a prior publication, the MCL35 risk assessment on 42 non-nodal samples was reported to correlate with overall survival [13].

MCL35 risk group stratification identifies low-, standard, and high-risk patients. The overall agreement between the MCL35 and Ki67 assays was just 37%, although there is a general overall correlation between the 2 types of data. Of interest, in our case series, the Ki67 proliferation rate appeared to err on the side of a higher risk category as compared with the MCL35 (the MCL35 identified more patients as low risk). Since the MCL35 assay is a multiplexed assay based on the weighted expression of 17 proliferation genes rather than a single marker, it may provide a more accurate reflection of the complex multifaceted/multifactorial nature of tumor biology and, as shown here and in prior publications, is highly reproducible as compared with other methods [9, 11]. Additional survival analyses are not available on this group of cases, which were selected for technical purposes.

Overall, the current data support the concept of molecular signatures, as assessed with digital gene expression profiling, as showing outstanding reproducibility and robust performance. The turnaround time for similar assays using the same platform in our clinical lab is, on average, 2–3 business days. As such, this assay may be an excellent choice for use in clinical trials wherein the relationships between biomarkers and patient outcome may lead to clinical practice changes. Recently, the MCL35 assay was successfully used in clinical trial of younger patients treated on Nordic MCL protocols and, along with MIPI, identified those patients with a dismal prognosis [15]. Additional studies evaluating the clinical utility of the MCL35 in older patients, including assessment of TP53 mutation status, are underway.

## Electronic supplementary material

ESM 1 (PDF 304 kb)

## Data Availability

Yes.

## References

[1] Cortelazzo S, Ponzoni M, Ferreri AJM, Dreyling M (2020). Mantle cell lymphoma. Crit Rev Oncol Hematol.

[2] Li JY, Gaillard F, Moreau A, Harousseau JL, Laboisse C, Milpied N, Bataille R, Avet-Loiseau H (1999). Detection of translocation t(11;14)(q13;q32) in mantle cell lymphoma by fluorescence in situ hybridization. Am J Pathol.

[3] Wiestner A, Tehrani M, Chiorazzi M, Wright G, Gibellini F, Nakayama K, Liu H, Rosenwald A, Muller-Hermelink HK, Ott G, Chan WC, Greiner TC, Weisenburger DD, Vose J, Armitage JO, Gascoyne RD, Connors JM, Campo E, Montserrat E, Bosch F, Smeland EB, Kvaloy S, Holte H, Delabie J, Fisher RI, Grogan TM, Miller TP, Wilson WH, Jaffe ES, Staudt LM (2007). Point mutations and genomic deletions in CCND1 create stable truncated cyclin D1 mRNAs that are associated with increased proliferation rate and shorter survival. Blood.

[4] Hoster E, Dreyling M, Klapper W, Gisselbrecht C, van Hoof A, Kluin-Nelemans HC, Pfreundschuh M, Reiser M, Metzner B, Einsele H, Peter N, Jung W, Wormann B, Ludwig WD, Duhrsen U, Eimermacher H, Wandt H, Hasford J, Hiddemann W, Unterhalt M, German Low Grade Lymphoma Study G, European Mantle Cell Lymphoma N (2008). A new prognostic index (MIPI) for patients with advanced-stage mantle cell lymphoma. Blood.

[5] Hoster E, Klapper W, Hermine O, Kluin-Nelemans HC, Walewski J, van Hoof A, Trneny M, Geisler CH, Di Raimondo F, Szymczyk M, Stilgenbauer S, Thieblemont C, Hallek M, Forstpointner R, Pott C, Ribrag V, Doorduijn J, Hiddemann W, Dreyling MH, Unterhalt M (2014). Confirmation of the mantle-cell lymphoma International Prognostic Index in randomized trials of the European Mantle-Cell Lymphoma Network. J Clin Oncol.

[6] Determann O, Hoster E, Ott G, Wolfram Bernd H, Loddenkemper C, Leo Hansmann M, Barth TE, Unterhalt M, Hiddemann W, Dreyling M, Klapper W, European Mantle Cell Lymphoma N, the German Low Grade Lymphoma Study G (2008). Ki-67 predicts outcome in advanced-stage mantle cell lymphoma patients treated with anti-CD20 immunochemotherapy: results from randomized trials of the European MCL Network and the German Low Grade Lymphoma Study Group. Blood.

[7] He JS, Chen X, Wei GQ, Sun J, Zheng WY, Shi JM, Wu WJ, Zhao Y, Zheng GF, Huang H, Cai Z (2019). Simplified MIPI-B prognostic stratification method can predict the outcome well-retrospective analysis of clinical characteristics and management of newly-diagnosed mantle cell lymphoma patients from China. Medicine (Baltimore).

[8] Klapper W, Hoster E, Determann O, Oschlies I, van der Laak J, Berger F, Bernd HW, Cabecadas J, Campo E, Cogliatti S, Hansmann ML, Kluin PM, Kodet R, Krivolapov YA, Loddenkemper C, Stein H, Moller P, Barth TE, Muller-Hermelink K, Rosenwald A, Ott G, Pileri S, Ralfkiaer E, Rymkiewicz G, van Krieken JH, Wacker HH, Unterhalt M, Hiddemann W, Dreyling M, European MCLN (2009). Ki-67 as a prognostic marker in mantle cell lymphoma-consensus guidelines of the pathology panel of the European MCL Network. J Hematop.

[9] Croci GA, Hoster E, Bea S, Clot G, Enjuanes A, Scott DW, Cabecadas J, Veloza L, Campo E, Clasen-Linde E, Goswami RS, Helgeland L, Pileri S, Rymkiewicz G, Reinke S, Dreyling M, Klapper W (2020). Reproducibility of histologic prognostic parameters for mantle cell lymphoma: cytology, Ki67, p53 and SOX11. Virchows Arch.

[10] Rosenwald A, Wright G, Wiestner A, Chan WC, Connors JM, Campo E, Gascoyne RD, Grogan TM, Muller-Hermelink HK, Smeland EB, Chiorazzi M, Giltnane JM, Hurt EM, Zhao H, Averett L, Henrickson S, Yang L, Powell J, Wilson WH, Jaffe ES, Simon R, Klausner RD, Montserrat E, Bosch F, Greiner TC, Weisenburger DD, Sanger WG, Dave BJ, Lynch JC, Vose J, Armitage JO, Fisher RI, Miller TP, LeBlanc M, Ott G, Kvaloy S, Holte H, Delabie J, Staudt LM (2003). The proliferation gene expression signature is a quantitative integrator of oncogenic events that predicts survival in mantle cell lymphoma. Cancer Cell.

[11] Scott DW, Abrisqueta P, Wright GW, Slack GW, Mottok A, Villa D, Jares P, Rauert-Wunderlich H, Royo C, Clot G, Pinyol M, Boyle M, Chan FC, Braziel RM, Chan WC, Weisenburger DD, Cook JR, Greiner TC, Fu K, Ott G, Delabie J, Smeland EB, Holte H, Jaffe ES, Steidl C, Connors JM, Gascoyne RD, Rosenwald A, Staudt LM, Campo E, Rimsza LM, Lymphoma/Leukemia Molecular Profiling P (2017). New molecular assay for the proliferation signature in mantle cell lymphoma applicable to formalin-fixed paraffin-embedded biopsies. J Clin Oncol.

[12] Robetorye RS, Ramsower CA, Rosenthal AC, Yip TK, Wendel Spiczka AJ, Glinsmann-Gibson BJ, Rimsza LM (2019). Incorporation of digital gene expression profiling for cell-of-origin determination (Lymph2Cx testing) into the routine work-up of diffuse large B cell lymphoma. J Hematopath (Google Scholars).

[13] Rauert-Wunderlich H, Mottok A, Scott DW, Rimsza LM, Ott G, Klapper W, Unterhalt M, Kluin-Nelemans HC, Hermine O, Hartmann S, Thorns C, Rymkiewicz G, Holte H, Dreyling M, Hoster E, Rosenwald A (2019). Validation of the MCL35 gene expression proliferation assay in randomized trials of the European Mantle Cell Lymphoma Network. Br J Haematol.

[14] Clot G, Jares P, Gine E, Navarro A, Royo C, Pinyol M, Martin-Garcia D, Demajo S, Espinet B, Salar A, Ferrer A, Muntanola A, Aymerich M, Rauert-Wunderlich H, Jaffe ES, Connors JM, Gascoyne RD, Delabie J, Lopez-Guillermo A, Ott G, Wright GW, Staudt LM, Rosenwald A, Scott DW, Rimsza LM, Bea S, Campo E (2018). A gene signature that distinguishes conventional and leukemic nonnodal mantle cell lymphoma helps predict outcome. Blood.

[15] Holte H, Beiske K, Boyle M, Trøen G, Blaker YN, Mykelbust J, Kvaløy S, Rosenwald A, Lingejærde OC, Rimsza LM, Smeland EB, Scott DW, Kolstad A (2018). The MCL35 gene expression proliferation assay predicts high-risk MCL patients in a Norwegian cohort of younger patients given intensive first line therapy. Br J Haematol.

