# Supplementary material for: Clinical laboratory validation of the MCL35 assay for molecular risk stratification of mantle cell lymphoma
Source: J Hematop. 2020 Oct 13;13(4):231–8. doi: 10.1007/s12308-020-00418-4 (PMC7661397; doi:10.1007/s12308-020-00418-4)
Supplement: Supplementary file 1 — (PDF 304 kb) [file 12308_2020_418_MOESM1_ESM.pdf]

Clinical laboratory validation of the MCL35 assay for molecular risk stratification of mantle cell lymphoma

Submitted to Journal of Hematopathology

Supplemental Table S1 All results

Validation ID	Total RNA Input (ng)	Norm	MCL35 Score	MCL35 Risk Group	Validation Cohort(s)	Ki67 (%)	Ki67 Risk Group
Val001-1	200	578	99.0	High Risk	Precision	N/A	N/A
Val001-2	200	632	89.1	High Risk	Precision	N/A	N/A
Val001-3	200	699	89.6	High Risk	Precision	N/A	N/A
Val001-4	200	694	99.5	High Risk	Precision	N/A	N/A
Val001-5	200	669	98.9	High Risk	Precision	N/A	N/A
Val001-6	50	190	90.2	High Risk	Precision	N/A	N/A
Val001-7	100	331	92.5	High Risk	Precision	N/A	N/A
Val001-8	200	660	83.6	High Risk	Precision	N/A	N/A
Val001-9	500	2138	88.4	High Risk	Precision	N/A	N/A
Val002-1	200	179	-127.4	Standard Risk	Precision	N/A	N/A
Val002-2	200	189	-124.8	Standard Risk	Precision	N/A	N/A
Val002-3	200	164	-131.8	Standard Risk	Precision	N/A	N/A
Val002-4	200	239	-123.0	Standard Risk	Precision	N/A	N/A
Val002-5	200	218	-134.0	Standard Risk	Precision	N/A	N/A
Val002-6	50	73	-77.1	Poor Quality	Precision	N/A	N/A
Val002-7	100	112	-122.7	Standard Risk	Precision	N/A	N/A
Val002-8	200	185	-134.1	Standard Risk	Precision	N/A	N/A
Val002-9	500	398	-132.3	Standard Risk	Precision	N/A	N/A
Val003-Tech1-1	200	789	-208.6	Low Risk	Precision	N/A	N/A
Val003-Tech1-2	200	638	-207.5	Low Risk	Precision	N/A	N/A
Val003-Tech1-3	200	751	-216.5	Low Risk	Precision	N/A	N/A
Val003-Tech1-4	200	681	-213.4	Low Risk	Precision	N/A	N/A
Val003-Tech1-5	200	1084	-208.8	Low Risk	Precision	N/A	N/A
Val003-Tech1-6	50	304	-216.8	Low Risk	Precision	N/A	N/A
Val003-Tech1-7	100	622	-212.4	Low Risk	Precision	N/A	N/A
Val003-Tech1-8	200	1128	-212.3	Low Risk	Precision	N/A	N/A
Val003-Tech1-9	500	1633	-216.9	Low Risk	Precision	N/A	N/A
Val003-Tech2-1	200	835	-198.1	Low Risk	Precision	N/A	N/A
Val003-Tech2-2	200	769	-200.7	Low Risk	Precision	N/A	N/A
Val003-Tech2-3	200	655	-206.3	Low Risk	Precision	N/A	N/A
Val003-Tech2-4	200	689	-199.1	Low Risk	Precision	N/A	N/A
Val003-Tech2-5	200	815	-202.7	Low Risk	Precision	N/A	N/A

Val003-Tech2-6	52	177	-186.2	Low Risk	Precision	N/A	N/A
Val003-Tech2-7	100	265	-183.4	Low Risk	Precision	N/A	N/A
Val003-Tech2-8	200	618	-192.5	Low Risk	Precision	N/A	N/A
Val003-Tech2-9	513.75	1554	-204.3	Low Risk	Precision	N/A	N/A
Val004-Tech1-1	200	1117	-15.7	High Risk	Precision	N/A	N/A
Val004-Tech1-2	200	1011	-8.4	High Risk	Precision	N/A	N/A
Val004-Tech1-3	200	1160	-8.8	High Risk	Precision	N/A	N/A
Val004-Tech1-4	200	1135	-9.4	High Risk	Precision	N/A	N/A
Val004-Tech1-5	200	1370	-9.0	High Risk	Precision	N/A	N/A
Val004-Tech1-6	50	345	-5.8	High Risk	Precision	N/A	N/A
Val004-Tech1-7	100	608	-8.7	High Risk	Precision	N/A	N/A
Val004-Tech1-8	200	839	-9.8	High Risk	Precision	N/A	N/A
Val004-Tech1-9	500	2565	-11.0	High Risk	Precision	N/A	N/A
Val004-Tech2-1	200	880	1.5	High Risk	Precision	N/A	N/A
Val004-Tech2-2	200	616	-2.1	High Risk	Precision	N/A	N/A
Val004-Tech2-3	200	727	-3.1	High Risk	Precision	N/A	N/A
Val004-Tech2-4	200	1066	-2.6	High Risk	Precision	N/A	N/A
Val004-Tech2-5	200	1023	-1.5	High Risk	Precision	N/A	N/A
Val004-Tech2-6	50	366	-3.3	High Risk	Precision	N/A	N/A
Val004-Tech2-7	100	670	-7.2	High Risk	Precision	N/A	N/A
Val004-Tech2-8	200	1177	-3.1	High Risk	Precision	N/A	N/A
Val004-Tech2-9	289.52	1562	-0.6	High Risk	Precision	N/A	N/A
Val005-Tech1-1	200	931	-116.9	Standard Risk	Accuracy, Precision	N/A	N/A
Val005-Tech1-2	200	1016	-119.8	Standard Risk	Accuracy, Precision	N/A	N/A
Val005-Tech1-3	200	1020	-115.2	Standard Risk	Accuracy, Precision	N/A	N/A
Val005-Tech1-4	200	1175	-117.6	Standard Risk	Accuracy, Precision	N/A	N/A
Val005-Tech1-5	200	1261	-119.3	Standard Risk	Accuracy, Precision	N/A	N/A
Val005-Tech1-6	64	469	-108.5	Standard Risk	Accuracy, Precision	N/A	N/A
Val005-Tech1-7	100	763	-114.0	Standard Risk	Accuracy, Precision	N/A	N/A
Val005-Tech1-8	200	409	-105.1	Standard Risk	Accuracy, Precision	N/A	N/A
Val005-Tech1-9	500	1258	-116.3	Standard Risk	Accuracy, Precision	N/A	N/A
Val005-Tech2-1	200	808	-109.1	Standard Risk	Accuracy, Precision	N/A	N/A

Val005-Tech2-2	200	738	-110.2	Standard Risk	Accuracy, Precision	N/A	N/A
Val005-Tech2-3	200	705	-100.8	Standard Risk	Accuracy, Precision	N/A	N/A
Val005-Tech2-4	200	852	-107.0	Standard Risk	Accuracy, Precision	N/A	N/A
Val005-Tech2-5	200	1048	-111.9	Standard Risk	Accuracy, Precision	N/A	N/A
Val005-Tech2-6	52	261	-88.6	Standard Risk	Accuracy, Precision	N/A	N/A
Val005-Tech2-7	100	434	-98.5	Standard Risk	Accuracy, Precision	N/A	N/A
Val005-Tech2-8	200	758	-114.1	Standard Risk	Accuracy, Precision	N/A	N/A
Val005-Tech2-9	280	1043	-106.1	Standard Risk	Accuracy, Precision	N/A	N/A
Val006-1	200	779	-200.1	Low Risk	Precision	N/A	N/A
Val006-2	200	841	-195.5	Low Risk	Precision	N/A	N/A
Val006-3	200	1083	-195.3	Low Risk	Precision	N/A	N/A
Val006-4	200	913	-195.7	Low Risk	Precision	N/A	N/A
Val006-5	200	1020	-201.4	Low Risk	Precision	N/A	N/A
Val006-6	50	178	-185.2	Low Risk	Precision	N/A	N/A
Val006-7	100	332	-173.4	Low Risk	Precision	N/A	N/A
Val006-8	200	583	-193.0	Low Risk	Precision	N/A	N/A
Val006-9	500	2762	-206.5	Low Risk	Precision	N/A	N/A
Val013	200	167	58.2	High Risk	Accuracy	N/A	N/A
Val014	200	943	50.7	High Risk	Accuracy	N/A	N/A
Val015	200	178	-109.3	Standard Risk	Accuracy	N/A	N/A
Val016	200	729	-106.5	Standard Risk	Accuracy, Precision	N/A	N/A
Val017	500	165	-222.9	Low Risk	Accuracy	N/A	N/A
Val018	200	432	-147.0	Low Risk	Accuracy	N/A	N/A
Val019	200	833	-149.0	Low Risk	Accuracy	N/A	N/A
Val020	200	248	-58.4	Standard Risk	Accuracy	N/A	N/A
Val021	200	243	-197.9	Low Risk	Accuracy	N/A	N/A
Val022	198.1	37	-91.0	Poor Quality	Accuracy	N/A	N/A
Val023	200	1470	93.0	High Risk	Accuracy	N/A	N/A
Val024	40	163	131.4	High Risk	Accuracy	N/A	N/A
Val025	200	327	269.7	High Risk	Accuracy	N/A	N/A
Val026	200	402	-159.0	Low Risk	FFPE Nodal	20	Standard Risk
Val027	414.4	38	-8.5	Poor Quality	B5 Nodal	20	Standard Risk
Val028	200	174	-87.0	Standard Risk	B5 Nodal	40	High Risk
Val029	200	1239	-118.1	Standard Risk	FFPE Nodal	40	High Risk
Val030	200	67	-117.9	Poor Quality	B5 Nodal	10	Standard Risk
Val031	200	276	-182.0	Low Risk	FFPE Nodal	10	Standard Risk
Val032	1290.8	49	75.3	Poor Quality	B5 Bone Marrow	70	High Risk
Val033	200	168	75.6	High Risk	B5 Bone Marrow	40	High Risk
Val034	445.2	22	105.5	Poor Quality	B5 Bone Marrow	30	High Risk
Val035	101.5	377	101.4	High Risk	FFPE Bone Marrow	60	High Risk
Val036	200	442	175.8	High Risk	B5 Bone Marrow	90	High Risk
Val037	200	5	-63.0	Poor Quality	FFPE Bone Marrow	5	Low Risk

Val038	1001.7	65	-21.6	Poor Quality	B5 Bone Marrow	5	Low Risk
Val039	111.3	16	-21.6	Poor Quality	FFPE Bone Marrow	5	Low Risk
Val040	200	204	169.0	High Risk	B5 Bone Marrow	30	High Risk
Val041	126.7	64	54.8	Poor Quality	FFPE Bone Marrow	5	Low Risk
Val042	200	735	-41.3	Standard Risk	Accuracy, Interfering Substances	N/A	N/A
Val042-DNA	200	675	-28.3	Standard Risk	Accuracy, Interfering Substances	N/A	N/A
Val042-Ethanol	200	46	-34.2	Poor Quality	Accuracy, Interfering Substances	N/A	N/A
Val042-FRN	200	20	-39.4	Poor Quality	Accuracy, Interfering Substances	N/A	N/A
Val042-limonene	200	460	-33.0	Standard Risk	Accuracy, Interfering Substances	N/A	N/A
Val042-RLT	200	83	-37.3	Standard Risk	Accuracy, Interfering Substances	N/A	N/A
Val043	200	2745	-52.7	Standard Risk	Accuracy, Interfering Substances	N/A	N/A
Val043-DNA	200	2535	-53.2	Standard Risk	Accuracy, Interfering Substances	N/A	N/A
Val043-Ethanol	200	992	-70.7	Standard Risk	Accuracy, Interfering Substances	N/A	N/A
Val043-FRN	200	363	-70.9	Standard Risk	Accuracy, Interfering Substances	N/A	N/A
Val043-limonene	200	1578	-45.9	Standard Risk	Accuracy, Interfering Substances	N/A	N/A
Val043-RLT	200	512	-76.7	Standard Risk	Accuracy, Interfering Substances	N/A	N/A
Val044	200	714	-173.5	Low Risk	Accuracy	N/A	N/A
Val045	200	411	-209.5	Low Risk	Accuracy	N/A	N/A
Val046	200	231	-66.9	Standard Risk	FFPE Extra Nodal	30	High Risk
Val047	200	150	-58.0	Standard Risk	B5 Extra Nodal	50	High Risk
Val048	200	172	-187.0	Low Risk	B5 Extra Nodal	10	Standard Risk
Val049	200	637	-188.9	Low Risk	FFPE Extra Nodal	15	Standard Risk
Val050	200	228	-181.9	Low Risk	FFPE Extra Nodal	5	Low Risk
Val051	836.5	63	-108.9	Poor Quality	B5 Extra Nodal	5	Low Risk
Val052	200	608	-154.0	Low Risk	Accuracy	N/A	N/A
Val053	200	1802	0.7	High Risk	Accuracy	N/A	N/A
Val054	200	307	-104.4	Standard Risk	Accuracy	N/A	N/A
Val055	200	305	-102.8	Standard Risk	Accuracy	N/A	N/A
Val056	200	792	13.7	High Risk	Accuracy	N/A	N/A
Val057	200	333	-54.8	Standard Risk	Accuracy	N/A	N/A
Val058	200	879	106.6	High Risk	Accuracy	N/A	N/A

Val059	200	392	-233.3	Low Risk	Accuracy	N/A	N/A
Val060	200	943	50.7	High Risk	Accuracy, Interfering Substances	N/A	N/A
Val060-DNA	200	736	97.1	High Risk	Accuracy, Interfering Substances	N/A	N/A
Val060-Ethanol	200	41	96.6	Poor Quality	Accuracy, Interfering Substances	N/A	N/A
Val060-FRN	200	18	154.4	Poor Quality	Accuracy, Interfering Substances	N/A	N/A
Val060-limonene	200	55	99.6	Poor Quality	Accuracy, Interfering Substances	N/A	N/A
Val060-RLT	200	37	131.9	Poor Quality	Accuracy, Interfering Substances	N/A	N/A
Val061	200	178	-109.3	Standard Risk	Accuracy, Interfering Substances	N/A	N/A
Val061-DNA	200	303	-61.6	Standard Risk	Accuracy, Interfering Substances	N/A	N/A
Val061-Ethanol	200	19	-114.8	Poor Quality	Accuracy, Interfering Substances	N/A	N/A
Val061-FRN	200	34	-71.4	Poor Quality	Accuracy, Interfering Substances	N/A	N/A
Val061-limonene	200	50	-116.2	Poor Quality	Accuracy, Interfering Substances	N/A	N/A
Val061-RLT	200	12	-43.4	Poor Quality	Accuracy, Interfering Substances	N/A	N/A
Val062	200	95	106.0	High Risk	B5 Extra Nodal	80	High Risk
Val063	200	374	120.0	High Risk	FFPE Extra Nodal	80	High Risk
Val064	200	24	-90.1	Poor Quality	B5 Extra Nodal	30	High Risk
Val065	200	40	-109.9	Poor Quality	FFPE Extra Nodal	10	Standard Risk
Val066	707	270	-96.3	Standard Risk	B5 Nodal	60	High Risk
Val067	200	445	-128.6	Standard Risk	Accuracy	N/A	N/A
Val068	200	43	-0.2	Poor Quality	Accuracy	N/A	N/A
Val069	200	488	-222.4	Low Risk	Accuracy	N/A	N/A
Val070	233	1145	-114.3	Standard Risk	Accuracy	N/A	N/A
Val071	200	554	-159.1	Low Risk	Accuracy	N/A	N/A
Val072	252	690	-123.3	Standard Risk	Accuracy	N/A	N/A
Val073	200	1016	-180.5	Low Risk	Accuracy	N/A	N/A
Val074	200	80	-161.8	Low Risk	Accuracy	N/A	N/A
Val075	200	793	-6.1	High Risk	Accuracy	N/A	N/A
Val076	200	674	-132.8	Standard Risk	Accuracy	N/A	N/A
Val077	200	299	-193.2	Low Risk	Accuracy	N/A	N/A
Val078	724.5	29	124.1	Poor Quality	B5 Extra Nodal	70	High Risk
Val079	200	192	67.0	High Risk	FFPE Extra Nodal	60	High Risk
Val080	200	27	120.9	Poor Quality	FFPE Extra Nodal	100	High Risk
Val081	200	29	116.4	Poor Quality	B5 Extra Nodal	80	High Risk
Val082	200	32	-94.3	Poor Quality	B5 Extra Nodal	30	High Risk

Val083	200	38	-12.2	Poor Quality	B5 Extra Nodal	60	High Risk
Val084	200	902	-153.9	Low Risk	FFPE Extra Nodal	50	High Risk
Val085	475.3	356	-99.9	Standard Risk	B5 Extra Nodal	40	High Risk
Val086	200	674	-132.8	Standard Risk	Accuracy, Interfering Substances	N/A	N/A
Val086-DNA	200	658	-78.5	Standard Risk	Accuracy, Interfering Substances	N/A	N/A
Val086-Ethanol	200	25	-90.2	Poor Quality	Accuracy, Interfering Substances	N/A	N/A
Val086-FRN	200	20	-98.7	Poor Quality	Accuracy, Interfering Substances	N/A	N/A
Val086-limonene	200	123	-104.1	Standard Risk	Accuracy, Interfering Substances	N/A	N/A
Val086-RLT	200	47	-115.0	Poor Quality	Accuracy, Interfering Substances	N/A	N/A
Val087	200	299	-193.2	Low Risk	Accuracy, Interfering Substances	N/A	N/A
Val087-DNA	200	629	-106.1	Standard Risk	Accuracy, Interfering Substances	N/A	N/A
Val087-Ethanol	200	77	-155.1	Poor Quality	Accuracy, Interfering Substances	N/A	N/A
Val087-FRN	200	20	-109.8	Poor Quality	Accuracy, Interfering Substances	N/A	N/A
Val087-limonene	200	90	-148.5	Low Risk	Accuracy, Interfering Substances	N/A	N/A
Val087-RLT	200	37	-132.1	Poor Quality	Accuracy, Interfering Substances	N/A	N/A
Val088	254.6	40	24.3	Poor Quality	FFPE Nodal	90	High Risk
Val089	200	550	-80.1	Standard Risk	FFPE Nodal	10	Standard Risk
Val090	200	1788	-215.0	Low Risk	FFPE Nodal	50	High Risk
Val091	200	202	54.0	High Risk	FFPE Nodal	95	High Risk
Val092	200	85	-51.2	Standard Risk	FFPE Nodal	70	High Risk
Val093	278.2	2179	32.2	High Risk	FFPE Nodal	80	High Risk
Val094	200	2076	-79.8	Standard Risk	FFPE Nodal	70	High Risk
Val095	226.8	18	-116.0	Poor Quality	FFPE Nodal	20	Standard Risk
Val096	200	70	-107.9	Poor Quality	FFPE Nodal	50	High Risk
Val097	347.7	76	-27.2	Poor Quality	FFPE Nodal	30	High Risk
Val098	200	131	13.1	High Risk	FFPE Nodal	50	High Risk
Val099	219.8	86	-137.5	Standard Risk	FFPE Nodal	10	Standard Risk
Val100	200	69	-105.1	Poor Quality	FFPE Nodal	30	High Risk
Val101	200	91	-124.8	Standard Risk	FFPE Nodal	30	High Risk
Val102	200	157	-79.8	Standard Risk	B5 Extra Nodal	50	High Risk
Val103	200	112	-64.8	Standard Risk	FFPE Extra Nodal	70	High Risk
Val104	200	44	-71.5	Poor Quality	FFPE Extra Nodal	N/A	No stain available
Val105	268.1	1970	-21.3	High Risk	FFPE Extra Nodal	80	High Risk
Val106	200	770	-191.1	Low Risk	FFPE Extra Nodal	10	Standard Risk

Val107	200	14	-6.8	Poor Quality	FFPE Extra Nodal	80	High Risk
Val108	200	27	-129.3	Poor Quality	FFPE Extra Nodal	40	High Risk
Val109	200	78	-109.7	Poor Quality	FFPE Extra Nodal	30	High Risk
Val110	220.5	346	-161.5	Low Risk	FFPE Extra Nodal	20	Standard Risk
Val111	200	609	-58.0	Standard Risk	FFPE Extra Nodal	50	High Risk
Val112	200	498	-207.9	Low Risk	FFPE Extra Nodal	30	High Risk
Val113	224.7	1109	66.2	High Risk	FFPE Extra Nodal	100	High Risk
Val114	200	1307	-225.5	Low Risk	FFPE Extra Nodal	20	Standard Risk
Val115	200	17	-56.8	Poor Quality	FFPE Extra Nodal	10	Standard Risk
Val116	73.5	58	-104.0	Poor Quality	FFPE Extra Nodal	20	Standard Risk
Val117	105.7	11	-48.9	Poor Quality	B5 Extra Nodal	15	Standard Risk
Val118	53.9	73	33.7	Poor Quality	FFPE Extra Nodal	80	High Risk
Val119	200	73	-188.5	Poor Quality	B5 Extra Nodal	10	Standard Risk
Val120	200	134	69.1	High Risk	B5 Extra Nodal	90	High Risk
Val121	71.4	7	-68.2	Poor Quality	B5 Extra Nodal	50	High Risk
Val122	102.9	938	-303.3	Low Risk	FFPE Extra Nodal	20	Standard Risk
Val123	200	203	-193.0	Low Risk	FFPE Extra Nodal	20	Standard Risk
Val124	200	1821	-167.2	Low Risk	FFPE Extra Nodal	30	High Risk
Val125	212.7	1081	-86.2	Standard Risk	B5 Extra Nodal	30	High Risk
Val126	200	160	-53.2	Standard Risk	B5 Extra Nodal	30	High Risk
Val127	200	1229	-156.7	Low Risk	FFPE Extra Nodal	50	High Risk
